# A deleterious variant of *INTS1* leads to disrupted sleep–wake cycles

**DOI:** 10.1242/dmm.050746

**Published:** 2024-08-27

**Authors:** Shir Confino, Yair Wexler, Adar Medvetzky, Yotam Elazary, Zohar Ben-Moshe, Joel Reiter, Talya Dor, Simon Edvardson, Gali Prag, Tamar Harel, Yoav Gothilf

**Affiliations:** ^1^ School of Neurobiology, Biochemistry and Biophysics, Faculty of Life Sciences, Tel-Aviv University, Tel-Aviv 6997801, Israel; ^2^ Pediatric Pulmonary & Sleep Unit, Hadassah Medical Center and Faculty of Medicine, Hebrew University of Jerusalem, Jerusalem 91120, Israel; ^3^ ALYN - Children and Adolescent Rehabilitation Center, Jerusalem 9109002, Israel; ^4^ Sagol School of Neuroscience, Tel-Aviv University, Tel-Aviv 6997801, Israel; ^5^ Department of Genetics, Hadassah Medical Center, Jerusalem 91120, Israel; ^6^ Faculty of Medicine, Hebrew University of Jerusalem, Jerusalem 9112102, Israel

**Keywords:** Integrator complex, INTS1, Sleep, Zebrafish, Circadian clock, Dopamine β-hydroxylase (DBH)

## Abstract

Sleep disturbances are common among children with neurodevelopmental disorders. Here, we report a syndrome characterized by prenatal microcephaly, intellectual disability and severe disruption of sleep–wake cycles in a consanguineous family. Exome sequencing revealed homozygous variants (c.5224G>A and c.6506G>T) leading to the missense mutations E1742K and G2169V in integrator complex subunit 1 (*INTS1*), the core subunit of the Integrator complex. Conservation and structural analyses suggest that G2169V has a minor impact on the structure and function of the complex, while E1742K significantly alters a negatively charged conserved patch on the surface of the protein. The severe sleep–wake cycles disruption in human carriers highlights a new aspect of Integrator complex impairment. To further study INTS1 pathogenicity, we generated Ints1-deficient zebrafish lines. Mutant zebrafish larvae displayed abnormal circadian rhythms of locomotor activity and sleep, as is the case with the affected humans. Furthermore, Ints1-deficent larvae exhibited elevated levels of dopamine β-hydroxylase (*dbh*) mRNA in the locus coeruleus, a wakefulness-inducing brainstem center. Altogether, these findings suggest a significant, likely indirect, effect of INTS1 and the Integrator complex on maintaining circadian rhythms of locomotor activity and sleep homeostasis across vertebrates.

## Teaser

**Summary:** A deleterious variant of INTS1, the core subunit of the Integrator complex, causes severe sleep disturbances in humans.

## INTRODUCTION

Precise activation and inhibition of RNA polymerase II (RNAPII) are crucial for transcriptional regulation in eukaryotes. The Integrator complex, which is associated with the C-terminal repeat domain of RNAPII, plays a pivotal role in the 3′-end processing of RNA molecules (Baillat et al., 2005; Pfleiderer and Galej, 2021; Beckedorff et al., 2020; Gardini et al., 2014). This evolutionary conserved multi-unit complex mediates the cleavage of nascent RNAs, enforcing transcriptional termination through an endonucleolytic activity of integrator complex subunit 11 (INTS11) (Welsh and Gardini, 2023; Dasilva et al., 2021; Elrod et al., 2019; Stadelmayer et al., 2014; Lai et al., 2015). RNA interference (RNAi)-mediated depletion of either INTS1 or INTS11 components in *Drosophila melanogaster*, *Caenorhabditis elegans* and the planarian *Schmidtea mediterranea* led to accumulation of unprocessed small nuclear RNA (snRNA) and blocked stimulus-dependent activation of snRNA transcription (Ezzeddine et al., 2011; Wu et al., 2019; Schmidt et al., 2018).

In humans, pathogenic variants of the gene encoding INTS1, the largest core component of the Integrator complex, are mostly located in the C-terminal region of the protein. These variants lead to severe developmental defects, including skeletal and facial dysmorphia, axial hypotonia, cataracts, and neurodevelopmental delay with lack of verbal communication and intellectual disability (OMIM:618571; Neurodevelopmental disorder with cataracts, poor growth, and dysmorphic facies) (Oegema et al., 2017; Krall et al., 2019; Zhang et al., 2020).

Here, we report a family with two siblings, who carry a predicted damaging variant resulting in a missense mutation within the C-terminal region of INTS1. These individuals exhibit a phenotype similar to those previously reported (Krall et al., 2019; Zhang et al., 2020), along with severely disrupted sleep–wake cycles. In mice, targeted disruption of the *Ints1* gene results in embryonic lethality at the early blastocyst stage (Krall et al., 2019; Hata and Nakayama, 2007), hindering the research on the roles of INTS1. However, zebrafish *ints1* knockouts have been reported to be viable at the larval stage (Krall et al., 2019), making the zebrafish an advantageous model for this disease and for studying INTS1 functions in vertebrates. Moreover, the zebrafish is a commonly used model in circadian clock and sleep research, with the advantage of being a diurnal species, i.e. one that is active during daytime (Vatine et al., 2011; Foulkes et al., 2016; Yokogawa et al., 2007). Therefore, to determine whether the sleep disturbances were directly related to deficient INTS1 function, rather than to an unrelated genetic defect or a secondary outcome of brain involvement, we generated and characterized Ints1-deficient zebrafish lines. Our results demonstrate the importance of INTS1 and, accordingly, the Integrator complex in circadian clock and sleep–wake regulation, partly through an indirect effect on norepinephrine production of the locus coeruleus (LC).

## RESULTS

### Clinical reports

**Fig. 1.**
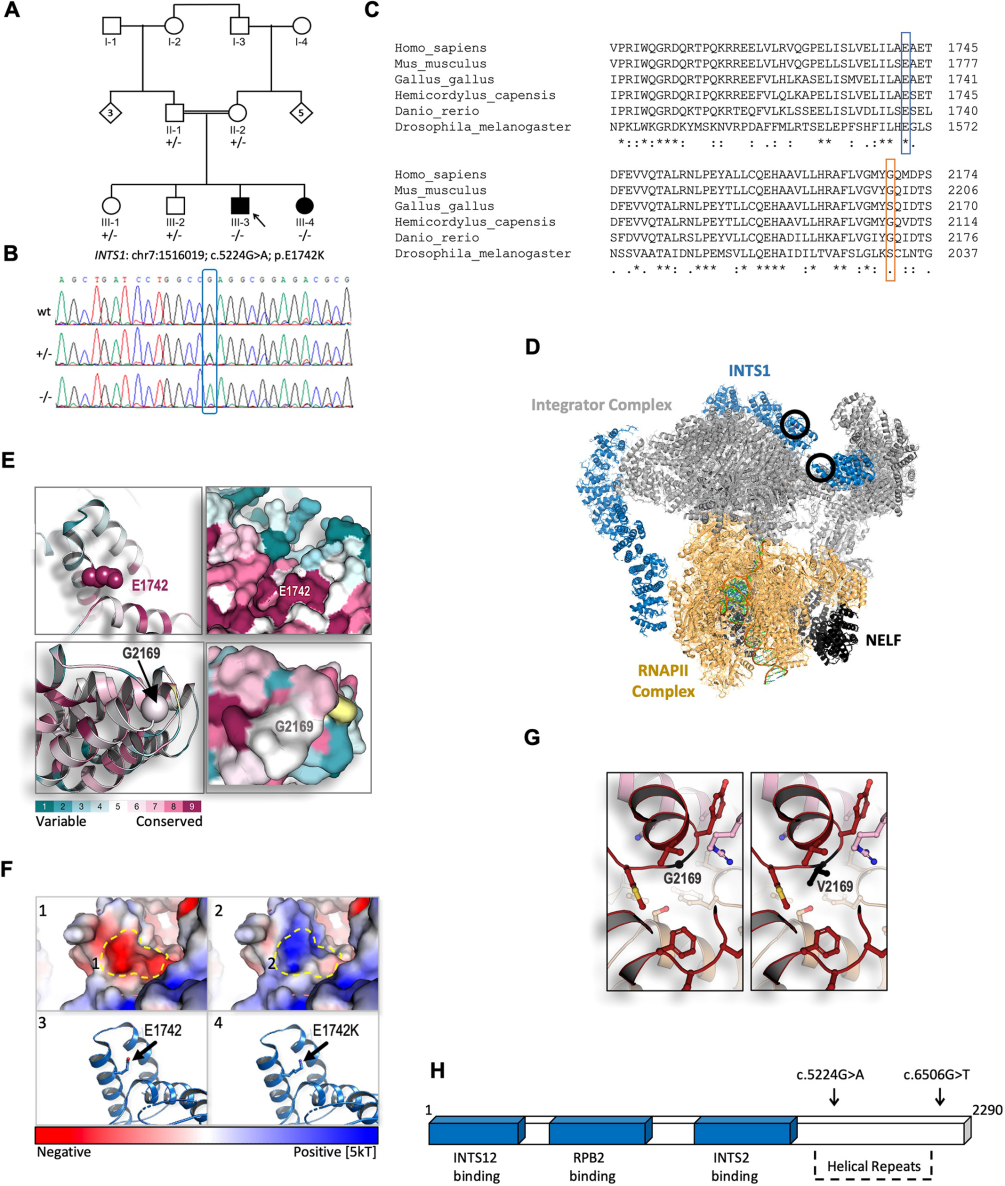
**Exome sequencing and structural modelling of *INTS1*.** (A) Pedigree of the family carrying the identified variant. Notice the parental consanguinity. (B) Sanger sequencing confirming the single nucleotide variant c.5224G>A (Gly→Ala) of *INTS1* in this family. (C) Predicted amino acid sequences surrounding the altered amino acid residues in the indicated species, demonstrating high evolutionary conservation of human Glu1742 (E1742) and less evolutionary conservation of human Gly2169 (G2169) when compared with the species indicated. (D) Protein structure of the Integrator complex during transcriptional regulation based on Fianu et al. (2021); Protein Data Bank accession code 7PKS (https://www.rcsb.org/structure/7PKS). Main complexes shown are the integrator complex subunit 1 (INTS1, blue); the Integrator subunits 2, 4-9 and 11 (Integrator complex, gray); DNA-directed RNA polymerase subunits β, E, F, RPB3, RPB7, RPB9, POLR2H, RPB10, RNAPII subunits D and K, RNAPII L2 domain-containing protein, and RPBI C-terminal domain peptide (RNAPII complex, gold); negative elongation factors A, B, C/D and E (NELF, black). Black circles indicate the position of the E1742K mutation (top circle) and the G2169V mutation (bottom circle). (E) Evolutionary structural conservation of human INTS1 variants, modelled using the Consurf algorithm (Ashkenazy et al., 2016). Regions surrounding E1742 and G2169 are shown as ribbon (left) and surface views (right). (F) Structural modelling of the E1742K substitution within the C-terminal region of INTS1. (1) Surface view of the conserved glutamic acid introduces a strong negatively charged pocket, indicated by the dashed line (in panels 1 and 2). (2) Surface view of the E1742K substitution. Exchanging the negatively charged E1742 to a positively charged K (Lys) alters the electrostatic dynamics from a strong negative to a strong positive one, predicting interference of surrounding structures. (3) Ribbon view showing the conserved glutamic acid residue. (4) Ribbon view showing the E1742K substitution within the most conceivable rotational isomer, i.e. rotamer, for K in this context. The electrostatic potential is represented by red-to-blue color spectrum, with potential values below −5 kT/e shown in red and values above +5 kT/e in blue; (potential was calculated by solving the Poisson-Boltzmann equation at 310K). (G) Structural modelling of the G2169V substitution. This mutation was modelled *in silico* using Coot and REFMAC5 (see Material and Methods ‘*In silico* analysis’). Ball and sticks structures of the WT (left) and mutated protein (right) are shown. (H) Schematic representation of INTS1 protein-interacting regions together with its C-terminal helical repeats tail. Sites of mutated amino acids are indicated by arrows above.

The proband (Fig. 1A, III-3) is the third of four children born to consanguineous first-cousin parents of Middle Eastern origin. He was born at 40 weeks gestation after an uncomplicated pregnancy, with a birth weight of 2720 g (z-score −1.92) and a head circumference of 32.5 cm (z-score −1.79). Congenital abnormalities included bilateral congenital cataracts, and moderate atrial and large ventricular septal defects on echocardiography, which were surgically closed at 3 months of age. He had significant hypotonia and did not meet any major developmental milestones – at the age of 6 years, he smiled, yet was nonverbal, could not crawl or sit and was wheelchair-bound. Seizures ensued at 6 months of age and were controlled with phenobarbital and levetiracetam. At 3 years of age, he was intubated owing to presumed aspiration pneumonia and required mechanical ventilation. A tracheostomy was placed and could not be removed later because of subglottic stenosis. At 4 years of age, he developed Hodgkin lymphoma and received chemotherapy, which led to remission. He was fed via a feeding gastrostomy. At 6 years of age, growth parameters were as follows: weight 14 kg (z-score −3.5) and head circumference 45 cm (z-score <−4). Physical examination showed plagiocephaly, hypertelorism, exotropia of the right eye, hyperpigmentation on the cornea, a broad nasal bridge, hirsutism of the back, axial hypotonia with significant head lag, laxity of the wrist and finger joints, and increased tone in the limbs with spasticity, scissoring of the lower limbs and increased deep tendon reflexes (DTRs). Brain MRI was normal.

The younger affected female sibling (Fig. 1A, III-4) had prenatal microcephaly apparent from 27 weeks gestation onwards. She was born at 39 weeks, with a birth weight of 2420 g (z-score −1.88) and head circumference of 30.5 cm (z-score −2.61). Similar to the proband, she had profound developmental delay, microcephaly, bilateral congenital cataracts and seizures. She had a bicuspid aortic valve. At 4 years and 9 months of age, her growth parameters were as follows: weight 14.5 kg (z-score −1.47), length 95 cm (z-score −2.47), head circumference 44 cm (z-score <−4). Dysmorphic features included hypertelorism, upslanted palpebral fissures, a broad nasal bridge, flat philtrum, missing front teeth due to premature loss of primary teeth, a high palate, a large hemangioma on the abdomen and a small hemangioma in the frontal area of the scalp, scoliosis, digit joint laxity, increased tone of the limbs and spasticity, and increased DTRs. Brain MRI results showed no abnormalities.

Importantly, the parents mentioned severe sleep disturbances in both affected children, suggesting a phenotypic expansion beyond the clinical manifestations previously reported (Oegema et al., 2017; Krall et al., 2019; Zhang et al., 2020) (Table S1).

### Exome sequencing and *in silico* analysis identify human *INTS1* variants that segregate with the disease

Exome sequencing identified two homozygous variants of *INTS1* (Table S2). The first variant – chr7:1516019[hg19]; NM_001080453.3; c.5224G>A; p.(Glu1742Lys) (Fig. 1A-B) – is rare (89 heterozygous and 0 homozygous carriers in the gnomAD v4.1.0 database), affects a conserved glutamic acid residue [Genomic Evolutionary Rate Profiling (GERP) score 5.12; Fig. 1C, top] and has been predicted to be damaging by several bioinformatic prediction tools [Consurf (http://consurf.tau.ac.il), SIFT (http://sift-dna.org), PolyPhen-2 (http://genetics.bwh.harvard.edu/pph2/index.shtml) and MutationTaster (https://www.mutationtaster.org/); CADD score 27.2 (https://cadd.gs.washington.edu/snv), AlphaMissense deleterious 0.826 (https://alphamissense.hegelab.org/)]. The second homozygous variant identified in *INTS1* – chr7:1510280[hg19]; NM_001080453.3; c.6506G>T; p.(Gly2169Val) – is also rare (0 carriers in the gnomAD database), affects a less-conserved amino acid (GERP score 5.49; Fig. 1C, bottom) and has lower pathogenic predictions [predicted to be tolerated by SIFT; CADD score 23.9; benign by REVEL, score 0.29, AlphaMissense uncertain (0.336)]. According to the American College of Medical Genetics and Genomics (ACMG) guidelines, both variants are classified as variants of uncertain significance (VUS) meeting criteria PM2 and PP1 (Richards et al., 2015). Since the two variants are linked in this family, the primary pathogenic variant driving the phenotype cannot be determined with full certainty. Both affected individuals are homozygous for the two variants, while the parents and two healthy siblings are heterozygous carriers.

### Structural modelling of the human INTS1 variant

Recent breakthroughs in the field of structural biology have made significant advancements in uncovering the overall structure of the Integrator complex, revealing stable heterodimeric interactions between its subunits and with additional complexes (Fianu et al., 2021; Zheng et al., 2020; Wu et al., 2017; Jia et al., 2021; Sabath et al., 2020). We utilized a recently published Cryo-EM structure (Fianu et al., 2021) to evaluate the INTS1 Glu→Lys (E1742K) and Gly→Val (G2169V) variants (Fig. 1D top and bottom circle, respectively), which shows that E1742 is exposed and accessible. To visualize the structural conservation of E1742, we conducted an analysis using ConSurf server (http://consurf.tau.ac.il) (Ashkenazy et al., 2016). Projection of the amino acid conservation on the INTS1 surface revealed that E1742 resides at the bottom of a highly conserved pocket, suggesting that this residue plays a substantial role, probably in the binding of another component to the complex (Fig. 1E, top). Representation of the calculated electrostatic surface potential [using Adaptive Poisson-Boltzmann Solver (APBS), https://apbs.readthedocs.io/en/latest/] indicates that the conserved pocket is negatively charged (Fig. 1F, top left). Therefore, a mutation to a positively charged residue, such as lysine, is predicted to disrupt the physicochemical properties of the pocket. We then modeled the structure of the E1742K mutated complex and recalculated the predicted electrostatic surface potential (Fig. 1F, top right). Although E1742 is not expected to interact with known proteins within the currently recognized Integrator complex (Fianu et al., 2021) (Fig. 1H) or induce a steric clash, because the residue is exposed a significant electrostatic shift is presumed to occur, switching from a negatively to a positively charged patch.

The G2169V mutation, however, appears to have no significant effect on the structure and function of the protein. We found that G2169, located in a loop connecting two helices, is not conserved (Fig. 1C,E, bottom), suggesting it does not have a significant structural role. Moreover, our structural analysis shows that there is sufficient space to accommodate valine at this position (Fig. 1G). The phi and psi dihedral angles of the glycine at the loop can be assumed by valine, predicting that this mutation is not deleterious. Nevertheless, as these two changes are linked, it cannot be determined with absolute confidence which of the two is the pathogenic variant that drives the phenotype.

### Individuals that are homozygous for the mutated *INTS1* allele exhibit disrupted daily sleep–wake cycles

**Fig. 2.**
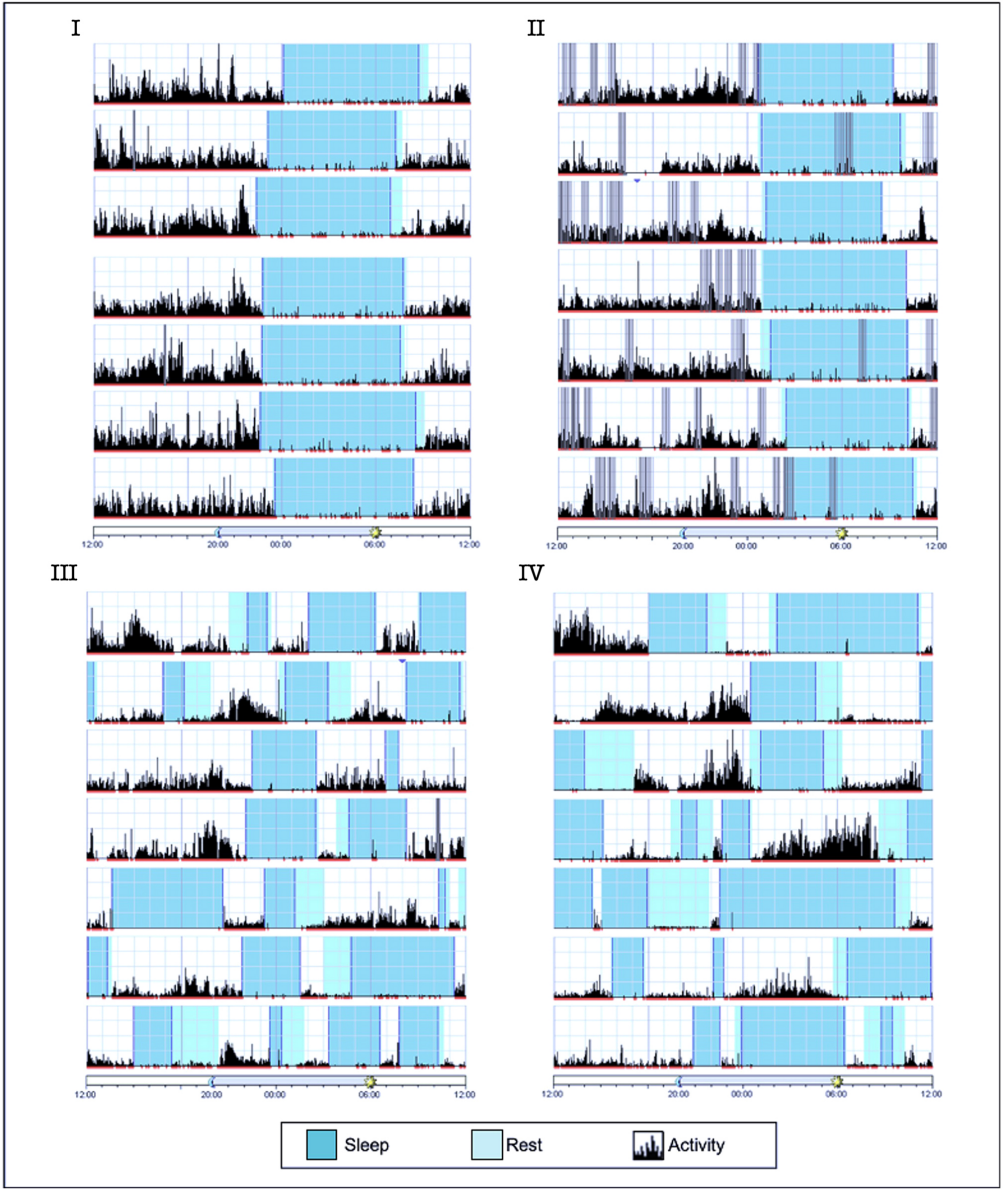
**Actigraphy of affected and healthy siblings demonstrating daily sleep arrhythmicity in individuals homozygous for the *INTS1* mutated allele.** Each horizontal segment of the actigraph depicts a full 24-h cycle from noon to the following noon. The height of the black lines on the chart reflects the extent and frequency of physical activity; blue and cyan depict sleep and rest time, respectively. Gray bars indicate invalid data. Actigraphs I and II of the healthy siblings (aged 10 and 8 years, respectively) show regular sleep patterns with a consistent sleep time and minor variations in waking time, while actigraphs III and IV of the affected children (aged 6 and 5 years, respectively) display several irregular sleep episodes and a substantial lack of daily rhythmicity.

For the assessment of sleep–wake regulation, both affected individuals, aged 6 and 5 years, wore actigraphs continuously on their wrists for 13 days. Actigraphy of individual III shown in Fig. 2, reveals irregular sleep patterns with total daily sleep lasting between 4 h 22 min and 10 h 26 min, and two to four bouts of sleep per day. Individual IV had a total of 4 h 8 min to 20 h 20 min of sleep per day, with two to four bouts of sleep per day. Additionally, actigraphy was done for 1 week for the two healthy heterozygous siblings aged 10 and 8 years, showing regular and mostly uninterrupted episodes of nighttime sleep lasting between 7 h 13 min and 8 h 6 min, respectively, and no daytime sleep. The sleep patterns of the affected children (III and IV) are consistent with the definition of irregular sleep–wake rhythm disorder (Sateia, 2014).

### Establishment of Ints1-deficient zebrafish lines

The well-preserved core structure of the INTS1 is highly conserved between human and zebrafish, with an almost identical protein length of 2190 and 2192 amino acids, respectively, and with 74% protein identity and 85% similarity (https://blast.ncbi.nlm.nih.gov/Blast.cgi). To further investigate Ints1 function, we utilized the CRISPR/Cas9 technology and generated *ints1* mutant zebrafish. By targeting exon 7 of the *ints1* gene, two mutant lines were prepared. The first comprises an 11-base pair (bp) deletion producing a frameshift and an early stop codon, resulting in a predicted 390-amino acid truncated protein. The second line has a single bp substitution, resulting in an immediate stop codon (K359X) and a truncated protein. Subsequent experiments on both *ints1* mutant lines revealed that the progeny genotypes of heterozygous intercrosses fit a Mendelian distribution. However, homozygous mutants die at advanced larval stages, after the absorption of the yolk sac and the commencement of exogenous feeding [10–14 days post-fertilization (dpf)], without reaching adulthood. Therefore, all succeeding experiments were conducted on the progeny of heterozygous fish intercrosses, followed by PCR-mediated genotyping.

### Ints1 is required for maintaining sleep–wake and locomotor activity cycles

**Fig. 3.**
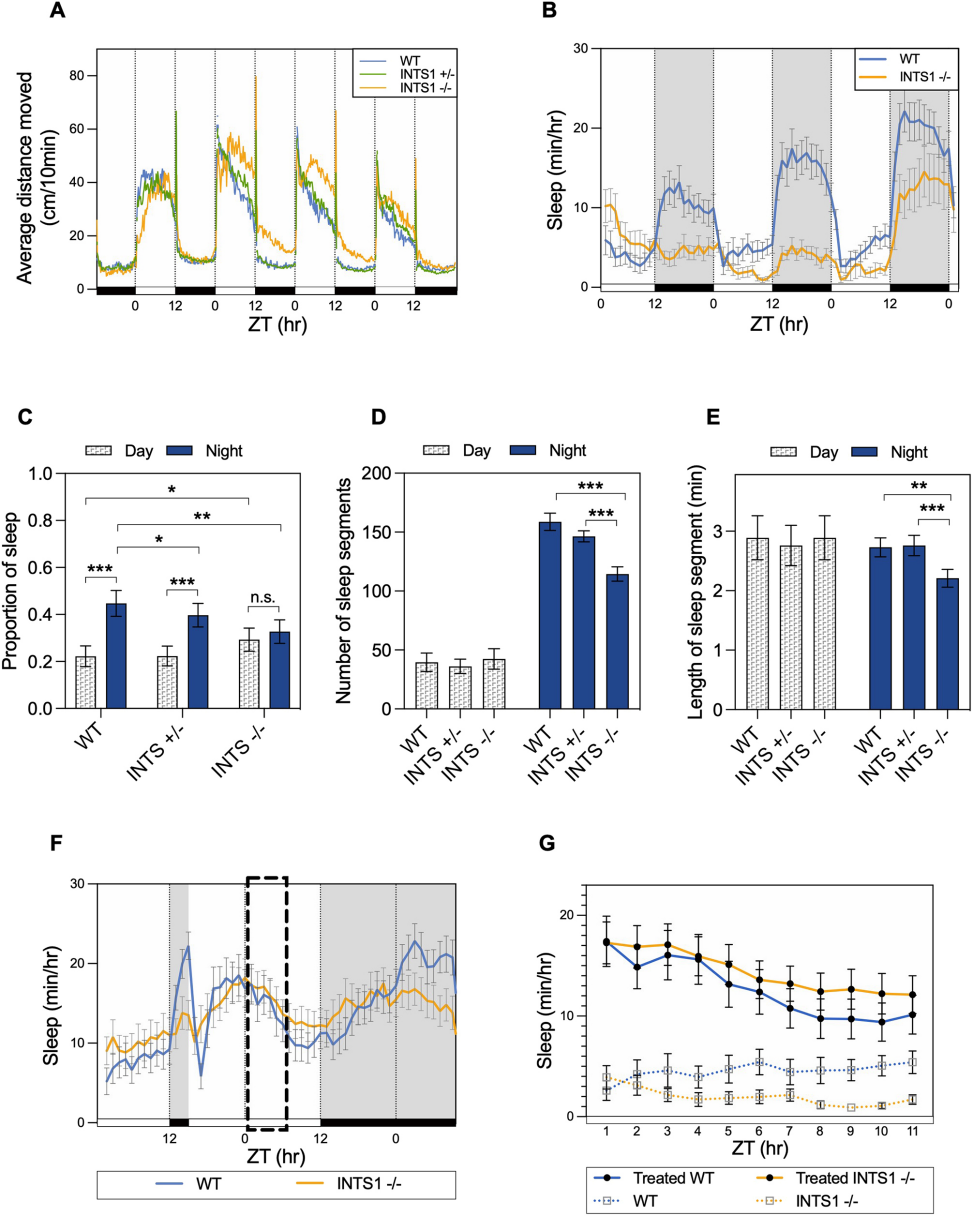
**Ints1 function is essential for maintaining locomotor activity and sleep–wake cycles in zebrafish.** (A) Locomotor activity analysis of *ints1* mutant larvae and their siblings under light/dark (LD) cycles. The average distance moved (cm/10 min) is plotted on the *y*-axis; the *zeitgeber* time (ZT, in hours; 0=lights on; 12=lights off) is plotted on the *x*-axis. White and black horizontal bars indicate light and dark conditions, respectively. *n*=83 homozygotes (INTS1 −/−); 169 heterozygotes (INTS1 +/−); 72 WT siblings. Progenies of heterozygous intercrosses were exposed to LD cycles and their locomotor activity was measured at 5–8 dpf. (B) Average sleep time of Ints1-deficient (INTS1 −/−) larvae and their WT siblings at 6–8 dpf under LD cycles. The average sleep time [min/h] is plotted on the *y*-axis; ZT (h) is plotted on the *x*-axis. White and black horizontal bars indicate light and dark conditions, respectively. Error bars represent ±s.e. (*n=*24 homozygotes; 24 WT siblings). (C) Sleep proportion separately computed under light and dark conditions using data from 6–8 dpf larvae. Both WT (*n*=72) and heterozygous (*n*=169) larvae exhibited a substantial difference in sleep proportion between day and night (***P<0.001, mixed model ANOVA using Type II Wald Chi-squared tests), while no significant difference was observed between day and night for homozygotes (*n*=83; error bars represent ±s.e.). Homozygous mutants exhibited significantly reduced nighttime sleep compared with WT and heterozygotes (**P<0.01 and *P<0.05, respectively, mixed model ANOVA using Type II Wald Chi-squared tests), and increased daytime sleep compared with WT (*P<0.05, mixed model ANOVA using Type II Wald Chi-squared tests). n.s., non-significant. (D) Number of sleep segments per night is reduced in Ints1-deficient larvae (*n*=83; ***P<0.001, mixed model ANOVA followed by post-hoc analysis) compared with WT (*n*=72) and heterozygous (*n*=169) larvae. Error bars represent ±s.e. (E) Reduced length of sleep segment during the night was observed for *ints1*-deficient larvae (*n*=83) compared with WT (*n*=72) and heterozygous (*n*=169) larvae (**P<0.01 and ***P<0.001, mixed model ANOVA followed by post-hoc analysis). Error bars represent ±s.e. (F) LD-trained larvae were exposed to 9 h of sleep deprivation, starting from 3 h after lights out. The average sleep time (min/h) is plotted on the *y*-axis; ZT (h) is plotted on the *x*-axis. White and black horizontal bars indicate light and dark conditions, respectively. Error bars represent ±s.e. (*n=*33 homozygotes; 38 WT siblings). Normal sleep rebound (indicated by dashed box) was measured in homozygotes with respect to their WT siblings. (G) Sleep rebound was measured in sleep-deprived WT and Ints1-deficient larvae compared with non-deprived larvae during the day after sleep-deprivation treatment. The average sleep time (min/h) is plotted on the *y*-axis; ZT (h) is plotted on the *x*-axis. Error bars represent ±s.e. (*n*=34 homozygotes; 32 WT siblings). Both genotypes exhibited a typical consolidated sleep pattern after the sleep-deprivation treatment.

Based on the prominently disrupted sleep–wake cycles observed in the affected siblings, we aimed to examine the influence of Ints1 deficiency on locomotor activity and sleep in zebrafish. Sleep in zebrafish larvae is defined by inactivity of >1 min or more (Zada et al., 2019, 2021; Rihel et al., 2010a). Accordingly, we monitored the locomotor activity and sleep pattern of *ints1* mutant larvae, and that of their wild-type (WT) and heterozygous siblings under 12-h light/12-h dark (LD) cycles. Homozygous larvae exhibited overall higher levels of locomotor activity compared with that of WT larvae, especially during the dark phase, and their peak of daytime activity was delayed by 4.2 h (Fig. 3A). The locomotor activity pattern of heterozygous larvae was similar to that of WT larvae (Fig. 3A). Sleep performance was calculated as the relative proportion of sleep during light–dark phase, revealing a significant reduction in sleep during the night and a relative increase during the day in homozygous mutants (Fig. 3B). Compared with homozygous mutants, significantly longer sleep duration at nighttime was observed in WT and heterozygous larvae; however, duration of night and day sleep was undistinguishable in Ints1 homozygous mutants (Fig. 3C). Furthermore, significant variation between genotypes was observed regarding nighttime sleep, with heterozygous larvae displaying intermediate sleep duration. During daytime, only homozygous larvae exhibited increased sleep behavior, while WT and heterozygotes showed comparable levels of sleep (Fig. 3C). Moreover, during the night, homozygous mutants exhibited a distinct reduction in sleep quantity, as revealed by reduced number (Fig. 3D) as well as shorter duration (Fig. 3E) of sleep segments, compared with those observed in their heterozygous and WT siblings. Altogether, these results indicate disturbed sleep homeostasis in Ints1-deficient larvae; they appear to be sleep-deprived and, therefore, sleep to a greater extent during daytime. WT zebrafish larvae manage sleep deprivation by a significant sleep rebound during the succeeding hours (Leung et al., 2019; Zhdanova, 2011). Accordingly, we examined how Ints1-deficient larvae respond to sleep deprivation. We exposed LD-entrained larvae to 9 h of sleep deprivation using mechanical tapping stimuli accompanied by constant exposure to nighttime light, starting 3 h after lights out. Prior to the sleep deprivation treatment, differences in sleep levels throughout the first 3 h of the night were observed; Ints1-deficient larvae slept significantly less than their WT siblings, possibly reflecting difficulties with sleep onset (Fig. 3F). During the hours following sleep deprivation, *ints1* mutants exhibited a sleep-rebound response similar to that of WT larvae (Fig. 3F, area encircled by dashed lines). The sleep-rebound data were compared with sleep data of regularly entrained larvae, indicating vastly elevated sleep duration following sleep deprivation for both genotypes (Fig. 3G). Altogether, these findings suggest that Ints1-deficient larvae exhibit a typical sleep rebound and that their increased daytime sleep appears to be a consequence of sleep deprivation during the night, probably due to difficulty initiating sleep during the initial dark-phase hours and maintaining it throughout the night.

### Ints1 is required for maintaining circadian rhythms of locomotor activity

**Fig. 4.**
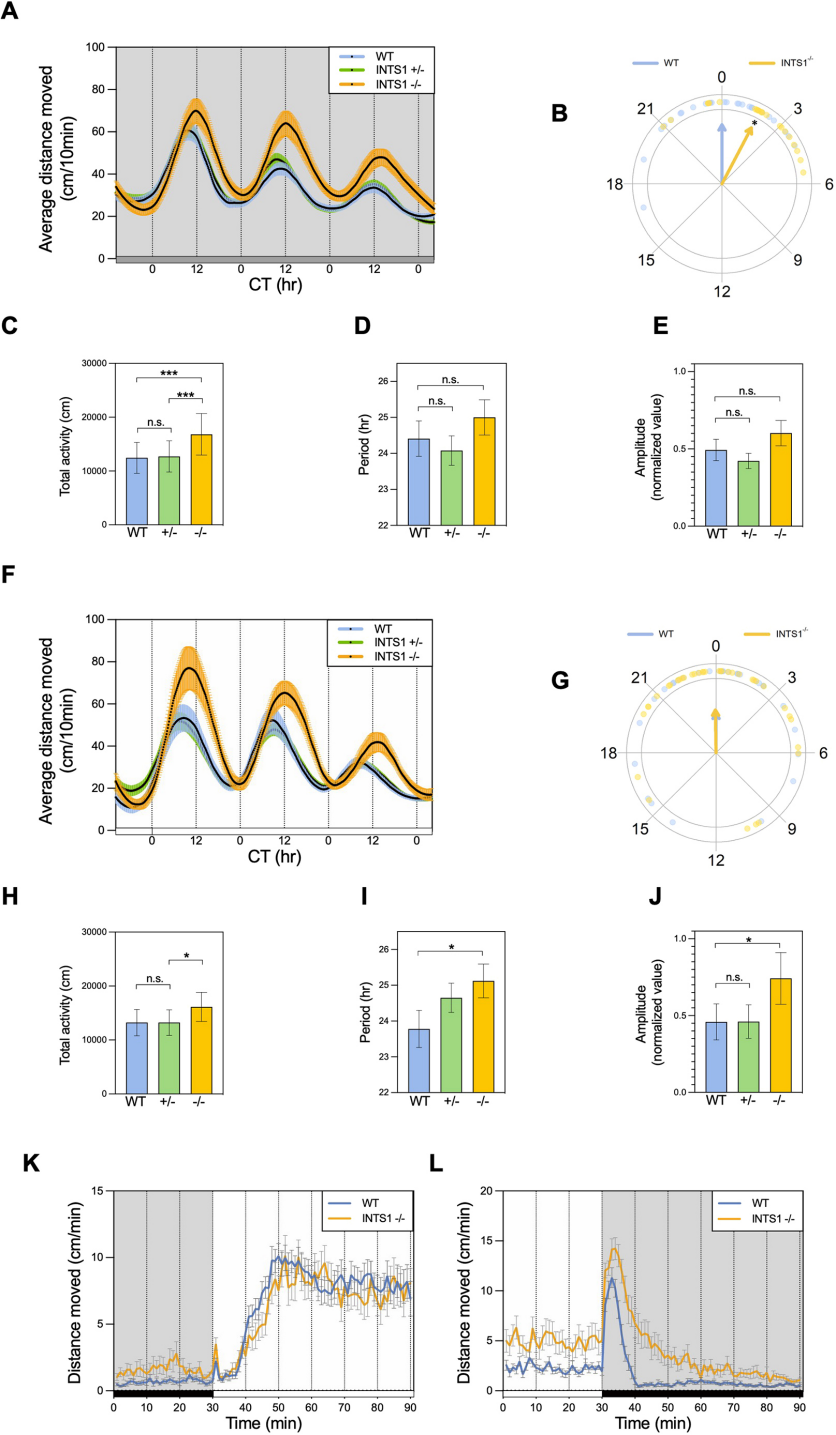
**Abnormal circadian rhythms of locomotor activity and dark-response in Ints1-deficient zebrafish larvae.** (A) Circadian locomotor activity of 6–8 dpf *ints1* homozygous (−/−) mutant larvae, and their heterozygous (−/+) and wild-type (+/+) siblings under constant dim light (DimDim, 7.5 lux). Progenies of heterozygous intercrosses were entrained under light/dark (LD) cycles for 3 days, followed by 2 days of light/dim light (LDim) cycles, and then exposed to DimDim for an additional 3 days, during which their activity was monitored. The *x*-axis represents the circadian time (CT, in hours); the *y*-axis represents the average distance travelled (cm/10 min). Colored areas represent the ±s.e. (*n*=31 homozygotes; 63 heterozygotes; 33 WT siblings). The gray horizontal bar at the bottom represents the illumination during monitoring, DimDim. (B) Circular plot depicting the phase of circadian locomotor activity rhythm of *ints1*-deficient (INTS1^−/−^) mutants (yellow) with respect to that of WT siblings (blue) under DimDim; each dot represents the value for one individual larvae. Arrow direction indicates the mean phase of each genotype, arrow length is inversely related to variation within the group. A 2-h phase-delay was observed for *ints1* mutants (*P<0.05, Watson-Williams test). (C–E) Under DimDim, Ints1-deficient larvae exhibit higher total locomotor activity (measured as distance moved in cm; ***P<0.001, mixed model ANOVA using Type II Wald Chi-squared tests) (C) with minor and insignificant variations in duration period (in hours) and amplitude (D and E, respectively) compared with WT larvae. n.s., non-significant; error bars represent ±s.e. (F) Circadian locomotor activity of 6–8 dpf *ints1* mutant larvae and their siblings under constant light (LL, 980 lux). Progenies of heterozygous intercrosses were entrained under LD cycles for 3 days, followed by 2 days of LDim cycles, and then exposed to LL for an additional 3 days, during which their activity was monitored. The *x*-axis represents the circadian time (CT, hours); the *y*-axis represents the average distance travelled (cm/10 min). Colored areas represent the ±s.e. (*n*=37 homozygotes; 67 heterozygotes; 35 WT siblings). The white horizontal bar at the bottom represents the illumination during monitoring, LL. (G–J) Under LL, Ints1-deficient larvae exhibit no differences in circadian phase compared to their WT siblings (G), but show significantly higher total activity (cm), longer period length (hours), and higher amplitude (*P<0.05 for all effects, mixed model ANOVA using Type II Wald Chi-squared tests). ‘n.s.’ denotes non-significant; error bars in H-J represent ±s.e. (K,L) Motor activity response of *ints1*-deficient larvae and their WT siblings to photic transitions, i.e. light-to-dark (K) and dark-to-light (L). LD-entrained larvae (5 dpf) were exposed to alternating light–dark conditions, and their activity was measured before and after the photic transition. The *x-*axis represents the time (min); the *y*-axis represents the average distance travelled (cm/min). Horizontal white and black bars (bottom) represent illumination during monitoring, i.e. light and dark, respectively. Error bars represent ±s.e. (*n*=33 homozygotes; 38 WT siblings). While no differences between genotypes were observed in response to the transition from dark to light (K), homozygous mutant larvae exhibited an irregular response to the transition from light to dark compared with their WT siblings, characterized by extended activity in response to darkness (L).

To test whether the disrupted rhythms of locomotor activity and sleep in *ints1* mutants resulted from an effect of the mutation on the circadian clock system, we analyzed the contribution of the intrinsic circadian clock of the larvae to the observed rhythmic behavior. Locomotor activity was examined under constant photic conditions at two levels of illumination: constant dim light (DimDim; 7.5 lux) or constant white light (LL; 980 lux). Under both light conditions, homozygous mutants exhibited elevated total activity relative to their siblings, with this effect being even more significant under DimDim. Moreover, a 2-h delay in the phase of the circadian activity rhythms was observed in homozygous mutants under these conditions. Under LL, homozygous mutant larvae also exhibited a longer period length and higher amplitude of circadian activity rhythms compared with those of their WT and heterozygous siblings (Fig. 4A-J). These results suggest that, on top of a generally elevated locomotor activity, circadian rhythms are affected by Ints1 deficiency in an illumination level-dependent manner.

### Ints1 is required for acclimation to darkness

The above data suggest that *ints1* mutant larvae are generally more active and sleep less than WT larvae, yet they undergo standard sleep homeostasis, as reflected by their sleep rebound after sleep deprivation. Therefore, we decided to investigate their general excitability. WT zebrafish larvae are known to respond to a sudden reduction in illumination with increased locomotor activity for a few minutes (Burgess and Granato, 2007; Blaser and Peñalosa, 2011). High-resolution analysis of the behavioral response to light–dark transition revealed an extended duration of this motor response in the *ints1* mutants (∼60 min compared with ∼10 min in WT siblings; Fig. 4K,L), indicating delayed acclimation to the dark environment. This effect was only observed in response to the light–dark transition, not the other way around, suggesting that the inability of *ints1* mutants to initiate sleep is likely due to a deficiency in adapt in the dark, characterized by a significantly prolonged activity bout when transferred to darkness compared with WT larvae.

### Ints1-deficient larvae exhibit significantly elevated gene expression levels of dopamine β-hydroxylase in the locus coeruleus

In search of a mechanistic explanation for the abnormal circadian rhythms of locomotor activity and sleep–wake cycles, we tested whether *ints1* mutants exhibit morphological deformations of the γ-aminobutyric acid (GABA) system. GABA is the principal inhibitory neurotransmitter in the vertebrate central nervous system. To test whether the *ints1* mutation has led to structural changes in the GABAergic system, we crossed heterozygous mutants (*ints^+/−^*) with transgenic fish in which the GABAergic neurons are fluorescently labeled [*TgBAC(gad1b:EGFP)*^nns25^] (Satou et al., 2013). Crossing heterozygous EGFP-positive transgenics with *ints^+/−^* fish resulted in a Mendelian distribution of genotypes with a fluorescently labeled GABAergic system. Comparison of the fluorescently labeled GABAergic systems among genotypes revealed no morphological differences (Fig. S1), eliminating abnormal neurogenesis of this system as a reason for the observed phenotype.

Another subcortical neuromodulatory system that plays a role in promoting wakefulness in vertebrates is the locus coeruleus (LC), a compact nucleus located deep within the brainstem. The LC serves as the source of an extensive noradrenergic neurotransmitter system and functions as a wakefulness-promoting center (Poe et al., 2020; Berridge and Waterhouse, 2003). Optogenetic stimulation of the LC in mice facilitates the transition from sleep to wakefulness, whereas suppression of LC activity decreases the duration of wakefulness (Carter et al., 2010, 2012). Pharmacological studies in zebrafish emphasize the conserved role of the noradrenergic system as a modulator of sleep–wake states (Berridge et al., 2012; Rihel et al., 2010b; Singh et al., 2015). The production of noradrenaline in the LC is determined by dopamine β-hydroxylase (Dbh). Knockout of the *dbh* gene, disruption of Dbh enzymatic activity, and ablation of Dbh-expressing LC neurons indicate that the LC noradrenergic system is necessary for modulating sleep and arousal in zebrafish larvae (Singh et al., 2015; Oikonomou and Prober, 2017; Cooper et al., 2005), as is the case in mammals (Berridge et al., 2012; Rihel et al., 2010b).

**Fig. 5.**
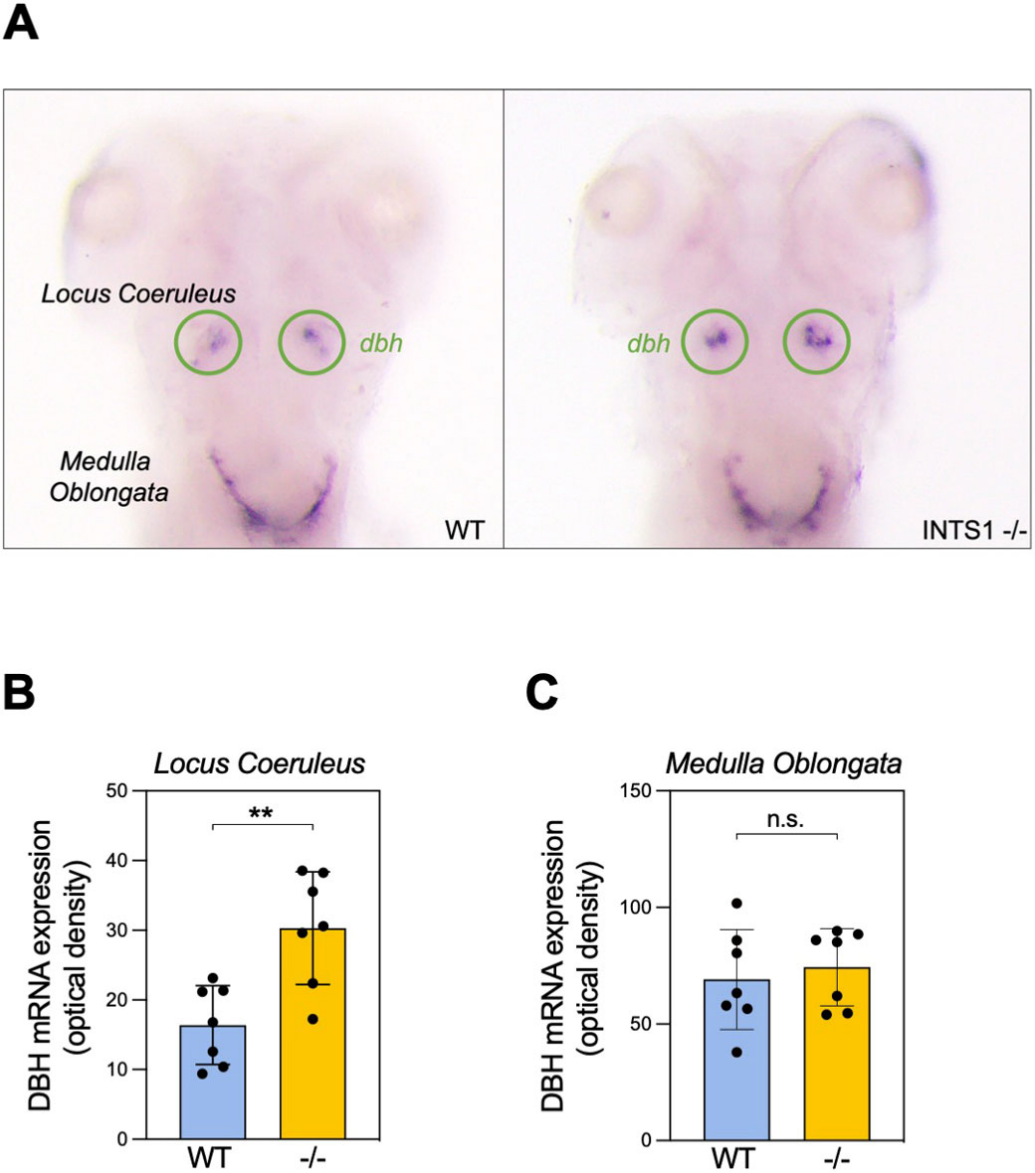
**Ints1-deficient zebrafish larvae exhibit increased *dbh* mRNA expression in the locus coeruleus but not in the medulla oblongata.** (A) Representative image, showing of *dbh* mRNA expression by whole-mount *in situ* hybridization of 5 dpf Ints1-deficient (right) and WT sibling (left) larvae (dorsal views). The locus coeruleus is encircled (green). (B) Quantification of the *dbh* mRNA signal in the locus coeruleus reveals significantly elevated expression in Ints1-deficient (−/−) larvae compared with their WT siblings (**P<0.01, two-tailed *t*-test assuming equal variance; *n*=7). (C) Quantification of *dbh* mRNA levels in the medulla oblongata shows no difference between Ints1-deficient larvae and their WT siblings (*n*=7). Error bars in B and C represent ±s.e.

Accordingly, to examine whether the effects of Ints1 on sleep are mediated by changes in the noradrenergic system, we monitored the levels and temporal distribution of *dbh* mRNA in *ints1* mutant and WT larvae by whole-mount *in situ* hybridization analysis. Two primary expression sites of *dbh* mRNA within the brain were identified: the LC and medulla oblongata (Fig. 5A). Quantification of the staining signal in both regions revealed substantially higher levels of *dbh* mRNA in the LC of *ints1* mutants compared with WT larvae, while no differences were observed in the medulla oblongata between genotypes (Fig. 5B,C). These findings imply that functional disruption of the LC noradrenergic system may be the cause of the sleep phenotypes observed in Ints1-deficient larvae.

## DISCUSSION

In the present study, we identified two homozygous *INTS1* variants that co-segregate with the phenotype in a consanguineous family with clinical manifestations consistent with the neurodevelopmental disorder with cataracts, poor growth, and dysmorphic facies (NDCAGF) [OMIM:618571]. One of the variants is probably pathogenic, whereas the other is in linkage disequilibrium with this variant. *INTS1* encodes the largest subunit of the Integrator protein complex, and is known to have a role in processing snRNAs and 3′ ends of coding genes (Baillat et al., 2005; Welsh and Gardini, 2023; Dasilva et al., 2021; Stadelmayer et al., 2014). As is the case for previously reported pathogenic INTS1 variants (Oegema et al., 2017; Krall et al., 2019; Zhang et al., 2020), the identified variants are sited in a highly conserved region at the C-terminus but not in regions that are thought to interact with other proteins of the currently known Integrator complex, such as INTS2, INTS12 or RPB2 (Fianu et al., 2021). As the *INTS1* E1742K variant does not seem to have a profound structural effect on INTS1 but only alters the electrostatic potential of an exposed surface of the pocket (Fig. 1F), we suggest that this patch may function in binding of an as-yet-unknown component of the complex.

Despite the similar clinical manifestations of the affected individuals to formerly described cases with pathogenic variants in *INTS1* (Table S1), sleep disorders linked with this gene or with Integrator complex dysfunction have not been reported up to now (Oegema et al., 2017; Krall et al., 2019; Zhang et al., 2020). To further establish this link in an animal model, we generated two zebrafish lines carrying full loss-of-function mutations in *ints1*. Characterization of these Ints1-deficient zebrafish reveals the crucial role of Ints1 in sleep and behavior among vertebrates. Our findings in zebrafish highlight a significant impairment in locomotor activity and sleep rhythms in relation to photic exposure. We demonstrated that during a light–dark transition, the mutants require an extended period of time to adjust to darkness. This effect was only observed for the light–dark transition, suggesting a potential impact on the onset of sleep. Examination of nighttime sleep patterns revealed that mutant larvae exhibit a reduced number of sleep segments and that these segments are significantly shorter than those detected in their siblings. Moreover, sleep analysis suggested impairment of sleep during the night, with consequent sleep rebound during the day. This may be further augmented by the slower adaptation to darkness and the, potentially, delayed sleep onset.

Since the Integrator complex is known to affect transcription initiation, transcription termination and alternative splicing of various genes (Dasilva et al., 2021; Elrod et al., 2019; Kirstein et al., 2021), the *ints1* mutation may influence many sleep-regulating pathways. Here, we showed the effect of Ints1 deficiency on the LC noradrenergic system, a brain-wide modulator of sleep and wakefulness (Carter et al., 2010; Singh et al., 2015; Oikonomou and Prober, 2017). We discovered significantly elevated expression levels of *dbh* mRNA in the LC – possibly reflecting an increased number of noradrenergic neurons due to developmental impairment within the LC – which both, potentially, influence sleep (Singh et al., 2015; Erwin and Radcliffe, 1993; Dyzma et al., 2010; Dzirasa et al., 2006). These findings warrant further investigation of LC neurons in *ints1* mutants, including molecular changes and functional activity, as well as the involvement of other neuronal systems in the sleep phenotype. In this regard, the zebrafish model may provide an advantage: utilizing available transgenic lines that express fluorescent proteins enables the visualization of different neurons, neuronal circuits and other non-neuronal brain cells in mutants compared with WT fish. Moreover, the use of transgenic lines that express calcium indicators would allow monitoring of neuronal activity in the LC and other brain regions.

The Ints1-deficient zebrafish, being a complete loss-of-function model, does not precisely mimic the deleterious human variants. Phenotypic rescue of *ints1* mutant fish by transgenic expression of human wild-type INTS1, compared with its deleterious variants, will provide more reliable evidence for the involvement of INTS1 and the affected brain regions in circadian clock and sleep regulation.

In summary, the combined data from humans carrying a missense *INTS1* variant and from Ints1-deficient zebrafish lines indicate the importance of this gene – and, therefore, the Integrator complex – in maintaining circadian rhythms of behavior and sleep homeostasis.

## MATERIALS AND METHODS

### Exome sequencing

After obtaining informed consent, exome analysis was conducted on DNA extracted from whole blood of the proband (Fig. 1A, III-3). Exonic sequences from DNA were enriched using the SureSelect Human All Exon 50 Mb V5 Kit (Agilent Technologies, Santa Clara, CA, USA). Sequences were generated on a HiSeq2500 system (Illumina, San Diego, CA, USA) as 125-bp paired-end runs. Read alignment and variant calling were performed using DNAnexus (Palo Alto, CA, USA) with default parameters and the human genome assembly hg19 (GRCh37) as reference. Exome analysis of the proband yielded 63 million reads, with a mean coverage of 107×. Variants were filtered out if they were off-target (>8 bp from splice junction), synonymous or showed minor allele frequency (<1%) in the gnomAD database.

### Sanger validation and segregation of the variant

Amplicons containing the *INTS1* variant were amplified using conventional PCR from genomic DNA of the proband, parents and siblings. Sequences were analyzed using the Sanger dideoxy nucleotide method.

### *In silico* analysis

Molecule inspection, rendering and modelling were performed using the molecular graphics application Coot REFMAC5 (Emsley et al., 2010) and PyMOL softwares (https://www.pymol.org) based on the published Cryo-EM structure (Fianu et al., 2021). The energy of point mutations was minimized using the *Idealisation* procedure with Refmac5 of the CCP4 suite. Evolutionary conservation analysis was carried out using Clustal Omega (https://www.ebi.ac.uk/jdispatcher/msa/clustalo), ConSurf (http://consurf.tau.ac.il/) and MULTIZ (https://genome.ucsc.edu/cgi-bin/hgTrackUi?db=mm9&g=multiz30way) algorithms (Ashkenazy et al., 2016; Sievers et al., 2011; Blanchette et al., 2004).

### Actigraphy and detection of sleep patterns in human patients

Following complaints from the parents regarding sleep disturbances, both affected individuals and two of their siblings received actigraphs (Actiwatch Spectrum Plus, Phillips Respironics, Andover, MA, USA) and wore them continuously on their wrists for 13 days. Data were then downloaded using the Actiwatch software package. The analysis included total sleep time for the major daily sleep duration, sleep latency (time to fall asleep), sleep efficiency (sleep duration out of time in bed) and general sleep patterns.

### Establishment of Ints1-deficient zebrafish lines

To establish the *ints1* mutant lines, the CRISPR-Cas9 system was utilized. An empty backbone of the pT7-gRNA zebrafish-optimized vector was obtained from Addgene (plasmid #46759), and two oligonucleotides were designed to target a specific sequence in the 7th exon of *ints1*, located on chromosome 3 of the zebrafish genome (5′-GGCCACCTGTGGTTATAAAG-3′; UCSC Genome Browser Database). The oligonucleotides were denatured at 95°C for 5 min, followed by progressive cooling to 4°C. The annealed oligonucleotides were then cloned into the pT7-gRNA plasmid with bacterial selection using ampicillin. The plasmid was then linearized using the BamHI restriction enzyme (New England Biolabs), followed by a standard phenol-chloroform purification procedure; sgRNA was synthesized using the T7 promoter for *in vitro* transcription (MAXIscript T7 Transcription Kit, AM1312, Invitrogen). The sgRNA (50 ng/µl) was mixed with Cas9 protein (1 μg/μl; TrueCut Cas9 Protein V2, A36498, Invitrogen) and microinjected into WT zebrafish embryos at the one-cell stage. Validation of microinjection efficiency was performed by DNA amplification from sampled 3 dpf F0 generation embryos (forward primer: 5′-CGCACTGTGTCTCGCATTTA-3′, Reverse primer: 5′-TGAGGCGGATCTTGATAAGG-3′), followed by enzymatic digestion with PsiI-v2 (New England Biolabs) that recognizes the 5′-TTATAA-3′ sequence in the WT allele (Fig. S2). Mosaic embryos were raised to adulthood and then crossed with WT fish to identify F1 generation mutants. A founder fish with an 11-bp deletion mutation was identified and crossed with a WT zebrafish. This mutation results in a frameshift after 358 amino acids, introducing an early stop codon following 32 off-frame amino acids. To decrease the potential off-targets risk, heterozygous F2 and subsequently F3 generations were outcrossed with WT zebrafish. The procedure was repeated with an additional founder fish carrying an A>T (Lys>Stop) point mutation, creating an immediate early stop codon and a truncated protein of 359 amino acids (Fig. S2). The two modified zebrafish lines were registered in the Zebrafish Model Organism Database (ZFIN; https://zfin.org/) as *Ints1^tlv13^* and *Ints1^tlv14^*, respectively. It should be noticed that, for both mutant lines, homozygous larvae did not reach adulthood and all experimental data were obtained using progeny form heterozygous intercrosses.

### Rhythmic locomotor activity and behavioral response to light–dark transition in zebrafish larvae

For monitoring of locomotor activity in zebrafish larvae, the DanioVision observation chamber (Noldus Information Technology, Wageningen, Netherlands) was used. Offspring from intercrosses of heterozygous *ints1* mutants were raised in an incubator under 12-h light (980 lux)/12-h dark cycles at 28°C. At 3 dpf, larvae were individually placed in wells of a 48-well plate and kept under 12-h light/12-h dim light (LDim; 980 lux and 7.5 lux, respectively) for an additional 2 days of habituation in the DanioVision chamber. Subsequently, larvae were exposed to various photic conditions for several days according to the experimental protocol, during which locomotor activity was recorded. Post-experiment DNA extraction was performed, followed by PCR to determine the genotype of each larva. All data were obtained using EthoVision 15.0 software (Noldus Information Technology, Wageningen, Netherlands) and analyzed for the distance moved by the larvae per time bin (cm/10 min). Experiments involving light–dark and dark–light transitions were conducted using a heightened time bin ratio (cm/1 min). Calculations of period length, amplitude, and phase of the circadian rhythm of locomotor activity, as well as total locomotor activity, were carried out as previously described (Ruggiero et al., 2021; Confino et al., 2022). Statistical differences in period, amplitude, and total activity between groups were determined by mixed model ANOVA using Type II Wald Chi-square tests. Statistical differences in the phase of activity were computed using the Watson-Williams test.

### Sleep analysis in zebrafish larvae

Zebrafish sleep analysis was conducted using the same principles as previously described (Zada et al., 2019, 2021; Confino et al., 2022), by calculating periods of inactivity (>1 min, with a stop velocity threshold of 0.59 cm/second and a start velocity of 0.60 cm/second) for each larva. Statistical analysis was based on linear mixed models, with the experimental trial and photic conditions (L, D or Dim) as random effects. *P*-values for fixed effects in mixed model ANOVA were obtained using Type II Wald Chi-square tests. The *P*-values of all post-hoc analyses were adjusted using the Benjamini-Hochberg (BH) procedure to control the false discovery rate (FDR) at the 0.05 level. Sleep deprivation was conducted on 5 dpf LD-entrained larvae by applying 9 h of mechanical tapping stimuli while maintaining continuous exposure to light. Significant differences in sleep segments between groups were assessed by mixed model ANOVA followed by post-hoc analysis.

### Whole-mount *in situ* hybridization

Whole-mount *in situ* hybridization assay and semi-quantification of *dbh* mRNA levels within the brain were performed as previously described (Confino et al., 2022; Vatine et al., 2009). Briefly, 5 dpf progeny from intercrosses of *Ints1^tlv13^* heterozygous fish (*n*=35 in each experiment; 2 repetitions) were fixed in 4% paraformaldehyde and kept overnight at 4°C, followed by PBTw washes and storage in 100% methanol at −20°C. Digoxigenin-labeled antisense probe for *dbh* mRNA (1 ng/1μl) was applied overnight at 65°C (the probe template was PCR amplified using forward primer 5′-GCGCTCTTTCCAGCTCACTGGATA-3′ and reverse primer 5′-CGAGCTTCAGATGGTAAAGGTGCAG-3′). Stained samples were placed in a 70% glycerol solution and observed using Olympus SZX16 microscope and Olympus DP74 digital camera. The integrated intensity of the staining signal (area in pixels multiplied by the average intensity) was quantified using ImageJ software (National Institute of Health, Bethesda, MD, USA) on an 8-bit transformed image with a subtracted light background (50), employing consistent quantification values for all samples (lower=0, upper=229). DNA extraction from fixed samples was performed using the REDExtract-N-AMP Tissue PCR KIT (Sigma-Aldrich), followed by PCR amplification and enzymatic digestion to determine the genotype of each sample. Statistical differences in staining levels were obtained using a two-tailed *t*-test, assuming equal variance.

### Fish and embryo maintenance

Adult zebrafish, heterozygous for the *ints1* mutations, were maintained in system water at 28°C under LD regime. Breeding was achieved by placing males and females (carrying identical genetic mutations) in a breeding tank with partition the evening prior to the spawning. Partition was removed the following morning as the lights were turned on. Eggs were collected and kept in Petri dishes containing embryo water with methylene blue (0.3 ppm) to prevent contamination. All husbandry and experimental procedures were approved by the Tel Aviv University Animal Care Committee (permit number TAU-LS-IL-2207-171-2) and performed in accordance with the requirements of the National Council for Animal Experimentations.

### Web resources

The ConSurf Web Server (http://consurf.tau.ac.il), UCSC Genome Browser (http://genome.ucsc.edu), ZFIN (https://zfin.org/), Ensembl (https://www.ensembl.org/index.html), Ensembl variation pathogenic prediction (https://www.ensembl.org/info/genome/variation/prediction/protein_function.html) and gnomAD (https://gnomad.broadinstitute.org/) were used.

## Supplementary Material

10.1242/dmm.050746_sup1 Supplementary information

Table S1. Clinical features of the reported individuals and comparison to the literature

Table S2. Rare homozygous variants that survived filtering
