# Supplementary material for: A deleterious variant of INTS1 leads to disrupted sleep–wake cycles
Source: Dis Model Mech. 2024 Aug 27;17(8):dmm050746. doi: 10.1242/dmm.050746 (PMC11381918; doi:10.1242/dmm.050746)
Supplement: Supplementary information [file dmm-17-050746-s1.pdf]

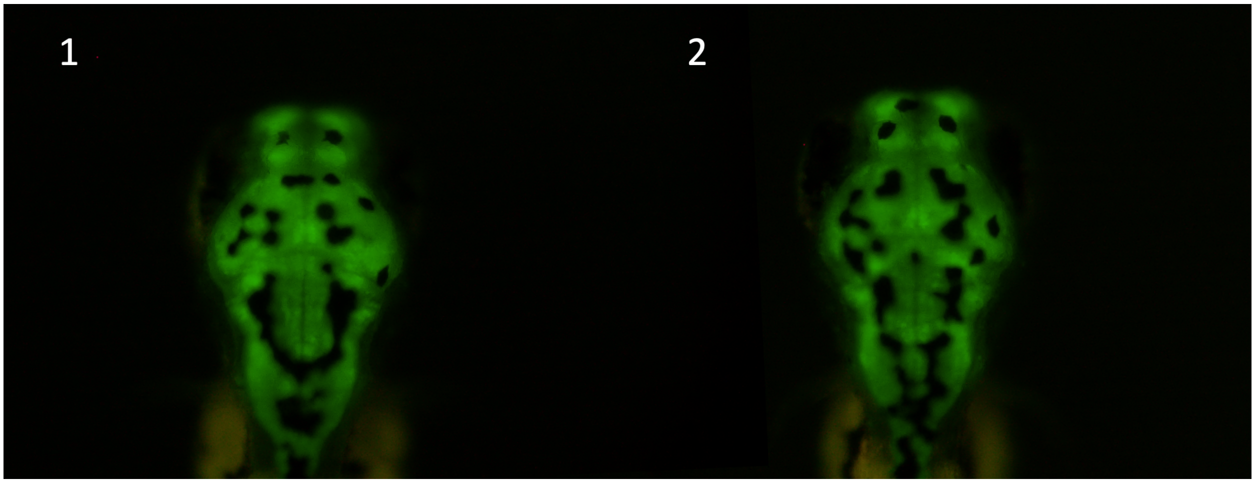

Fig. S1. Ints1-deficient larvae display no morphological deformations in the GABAergic system. In an attempt to identify a mechanistic explanation for the disrupted circadian clock and sleep phenotypes, the zebrafish transgenic line *TgBAC(gad1b:EGFP)*^{nns25} was utilized. Heterozygous *ints1*-mutant fish carrying a single allele of the transgenic insertion were crossed with fish heterozygous for the *ints1* mutation. The resulting progeny, positive for *TgBAC(gad1b:EGFP)*^{nns25} and exhibiting Mendelian distribution of the mutation, were inspected at the age of 3 dpf. No morphological abnormalities in the GABAergic system were observed in the mutant larvae. Representative WT (1) and *ints1*$^{-/-}$ (2) 3 dpf larvae (dorsal view), positive for *gad1b:EGFP*, are shown.

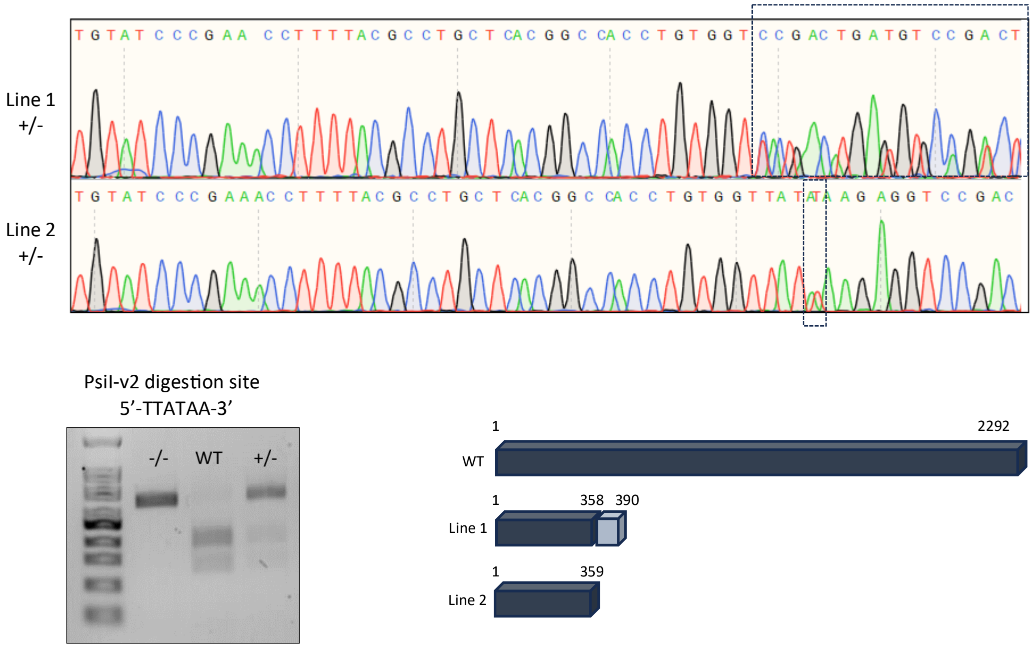

Fig. S2. Zebrafish *ints1* mutated lines.

Top. Sanger sequencing of a heterozygote fish from each *ints1*-mutant line demonstrating the mutated genomic regions. Dashed boxes designate the double sequencing peaks.

Bottom left. Electrophoresis gel analysis of PCR products amplified from WT and *ints1*-mutant genomic DNAs (using the primer set 5'-CGCACTGTGTCTCGCATTTA-3' and 5'-TGAGGCGGATCTTGATAAGG-3'', yielding a 622 bp product), followed by enzymatic digestion with PsiI-v2, which recognizes a specific sequence within the WT allele.

Bottom right. Schematic representation of the Ints1 mutated protein of each line compared with the WT protein. Dark blue boxes indicate the WT protein sequence; the grey box represents modified amino acids due to a frameshift within the coding sequence resulting from the 11bp deletion.

Disease Models & Mechanisms • Supplementary information

Table S1. Clinical features of the reported individuals and comparison to the literature

Available for download at
https://journals.biologists.com/dmm/article-lookup/doi/10.1242/dmm.050746#supplementary-data

Table S2. Rare homozygous variants that survived filtering

Available for download at
https://journals.biologists.com/dmm/article-lookup/doi/10.1242/dmm.050746#supplementary-data
